# ID 210 - Vacinação contra a Covid-19 no Brasil: descrição dos erros de imunização na nova tecnologia incorporada pelo Sistema Único de Saúde, 2021-2024, Brasil.

**DOI:** 10.5327/2237-9622.2025.v34s1.210

**Published:** 2025-11-25

**Authors:** Paulo Henrique Santos Andrade, Roberta Mendes Abreu Silva, Soniery Almeida Maciel, George Ricardo dos Santos, Krishna Mara Rodrigues Freire, Carla Dinamerica Kobayashi, Martha Elizabeth Brasil da Nóbrega, Monica Brauner de Moraes, Cibelle Mendes Cabral, Felipe Daniel Cardoso, Adriano Ferreira Martins, Jadher Percio

**Keywords:** erros de medicação, vacinas contra covid-19; farmacovigilância, avaliação da tecnologia biomédica

## Abstract

**Introdução:**

A introdução das vacinas contra a covid-19, como as de mRNA e vetor viral, no Sistema Único de Saúde (SUS) representou uma inovação significativa em saúde pública, mas também trouxe desafios relacionados ao gerenciamento seguro dessas novas tecnologias. A análise de erros de imunização (EI) visa fornecer dados reais, que possam informar gestores e administradores sobre a segurança e os ajustes necessários na implementação dessas vacinas, contribuindo para a prospecção tecnológica e para o desenvolvimento de estratégias de mitigação eficazes no âmbito da farmacovigilância.

**Método:**

Estudo descritivo realizado no âmbito das ações de farmacovigilância de vacinas no País, baseado nas notificações de erros de imunização (EI) relacionados às vacinas covid-19, registradas entre 17 de janeiro de 2021 e 17 de janeiro de 2024 no e-SUS Notifica, sistema de informações do Ministério da Saúde. O desfecho principal foi a ocorrência de EI, e o secundário a ocorrência de evento supostamente atribuído à vacinação ou imunização (Esavi) classificado como A3 (reação relacionada a EI). A análise descritiva dos dados foi executada no software R Studio-4.3.1. Foram calculados os coeficientes de incidência de EI por doses administradas (DA) segundo semana epidemiológica, características sociodemográficas, estado gestacional, vacina e tipo de erro.

**Resultados:**

Foram notificados 70.293 EI após 517.276.512 doses administradas (12,8/100.000 DA). As maiores incidências de EI foram encontradas para a faixa etária de 12 a 17 anos (31,3 erros/100.000 DA), vacina CoronaVac (18,5 /100.000 DA) e no estado de Alagoas (41,1 /100.000 DA). Entre as gestantes, o coeficiente de incidência de EI foi de 88,7/100.000 DA, relacionados, em sua maioria, à exposição durante a gravidez, à administração de vacina incorreta e à vacina AstraZeneca. Entre os EI, em geral, destacaram-se: "Administração de vacina incorreta" (2,9/100.000 DA), "Utilização de vacina vencida" (2,4/100.000 DA), "Administração de vacina para idade inadequada" (2,3/100.000 DA). Do total de EI notificados, 95 (1,8%) apresentaram algum Esavi classificado como A3 (<0,1/100.000 DA), sendo a maioria (98,9%) não grave.

**Conclusão:**

Os EI foram mais frequentes entre crianças e adolescentes e entre as vacinas utilizadas nessa faixa etária, configurando-se como um grupo prioritário para a implementação de ações destinadas à redução dos EI no País. Esses erros são compreensíveis em um cenário de rápida implementação de novas tecnologias vacinais, mas reforçam a importância de capacitar continuamente os profissionais de saúde. A adoção de medidas proativas é essencial para minimizar os EI e assegurar a confiança e a segurança da vacinação, especialmente em contextos de inovação tecnológica e urgência sanitária.

